# Enhancement of Medical Students' Performance and Motivation in Pathophysiology Courses: Shifting From Traditional Instruction to Blended Learning

**DOI:** 10.3389/fpubh.2021.813577

**Published:** 2022-01-26

**Authors:** Dan Wang, Junhai Zhou, Qiuhui Wu, Guannan Sheng, Xin Li, Huiling Lu, Jing Tian

**Affiliations:** ^1^Education Evaluation and Faculty Development Center, Guilin Medical University, Guilin, China; ^2^Department of Foreign Languages, Guilin Medical University, Guilin, China; ^3^Department of Pathophysiology, Guilin Medical University, Guilin, China; ^4^Department of Mathematics and Physics, Guilin Medical University, Guilin, China; ^5^Department of Physiology, Guilin Medical University, Guilin, China

**Keywords:** education, medical students, local university, blended learning, MOOC, pathophysiology

## Abstract

Blended learning is a learning approach that combines face-to-face classroom lectures and e-learning. It has grown rapidly to be commonly used in medical institutions, especially in the local medical universities where there is lack of qualified teachers and instructional materials. Massive open online courses (MOOCs) are the latest revolution in e-learning and provides learners with access to quality educational resources. Nevertheless, there is seldom reports concerning how to effectively integrate MOOCs into blended learning in local universities, as well as the evaluation of knowledge outcomes. In order to achieve this aim, a blended learning approach was carried out in teaching pathophysiology in Guilin Medical University. This blended learning model was based on combination of Chinese University MOOC with case based learning (CBL), as an alternative to conventional learning. The medical students in the 2017 and 2018 classes received the blended learning method, while the medical students in the 2015 class received the traditional classroom instruction. The results showed that students in the 2017 and 2018 performed significantly better than students in the 2015 class at mid-term exam and the final exam. Perception surveys also revealed that both students and teachers had positive attitude toward blended learning, and they shared similar viewpoints of blended learning. A large proportion of students and teachers believed that the blended learning enhanced students' motivation to learn independently, improved their time management skills, and allowed them to experience personalized learning. Also, most students and teachers recognized that Chinese University MOOC provided substantial educational resources suitable for their need. In addition, teachers indicated that the blended learning improved student learning quality, facilitated interaction between teachers and students, and helped them to establish a student-centered model in teaching pathophysiology. Overall, the blended learning method that combines Chinese University MOOC with CBL is effective in enhancing students' achievement and motivation in pathophysiology than the traditional learning method, and helps to strengthen the cultivation of talent in local medical universities.

## Introduction

With the development of internet and information technology, e-learning have become popular in medical education. Generally, e-learning refers to the use of new multimedia and communication technologies to enable the delivery of part or all of a course (1). This online learning setting is able to transcend the boundaries of institutions, time and geographical location. It allows students to learn at their own rate and plan in order to obtain a satisfactory learning outcome, due to their availability and ease of access to a large amount of learning resources (2). Therefore, e-learning is becoming more and more important in medical institutions of higher education. Nevertheless, e-learning also has some disadvantages (3). First, the high cost of technical infrastructure and network equipment is the most important barrier to e-learning development. Secondly, students are encouraged to depend on themselves during the online study, while the lack of strong intrinsic motivation and high time management skills may decrease student's learning effectiveness. In addition, when it comes to communication between students and teachers, face-to-face interaction in traditional methods may be more effective than e-learning.

Actually, many local universities are still being based on traditional learning method and follow the conventional setting of face-to-face lectures in a classroom instead of e-learning. Unlike e-learning, traditional learning requires students to attend classes at fixed time and place, which may facilitate the development of time management skills (4). Also, when students go to class, they have to learn how to interact with teachers and peers from diverse backgrounds and cultures. It is helpful to strengthen student's social skill and broaden their horizons. On the other hand, traditional learning is constrained by classroom size and teacher-student ratio. Undoubtedly, this constraint is more pronounced in local medical universities in the developing countries, where the school size and the number of qualified teachers has not increased in proportion to the rapid increase in school enrollments (5).

Due to the merits and demerits of both traditional learning and e-learning, their careful combination would be better than replacing each other. Blended learning is one of modern learning that combines face-to-face traditional learning and online learning. There is increasing evidence that blended learning is more effective in knowledge acquisition than traditional learning (6, 7). Consequently, a growing number of institutions of higher education are adopting blended learning including Guilin Medical University.

Recently, massive open online courses (MOOC) is developing rapidly and widely used in education. The aim of MOOC is to provide open and high-quality online education resources for self-study, and thus undergoes a shift from teacher-centered to student-centered learning (8). In China, MOOC construction began in 2013 and has made great strides in recent years. Until now, these platforms have been available online including Chinese University MOOC, Tsinghua University's “School Online,” Shanghai Jiaotong University's “Good University Online,” and the domestic basic education MOOC platform such as Zhihuishu and Fanya (9). Among them, Chinese University MOOC is the largest online open education platform, and provides the public with high-quality courses in science, medicine, engineering, economic and computer from the top universities in China (10). An increasing number of reports have indicated that Chinese University MOOC has made big contributions to the development and reform of higher education in China and the world (11).

In this study, Chinese University MOOC platform combined with classroom teaching was applied to pathophysiology courses for 3rd-year medical students in Guilin Medical University. Moreover, we here implemented case based learning (CBL) as an alternative to traditional formal learning. The use of blended learning based on Chinese University MOOC and CBL was compared with the traditional classroom method. Meanwhile, the reaction of our students and our faculty to these reform initiatives was explored. The goal of this study is to find a approach to improve the quality and outcomes of pathophysiology teaching suitable for the local medical universities.

## Methods

### Students

In Guilin Medical University, students take the pathophysiology courses in their 3rd year. The students who participated in this study were undergraduate clinical students in the academic years 2015, 2017, and 2018. Four hundred and thirty-one students from the 2015 class who attended traditional lectures were selected as control group (traditional learning methods). Four hundred and eighteen students from the 2017 class and 586 students from the 2018 class attended the blended learning courses that combined Chinese University MOOC resources with CBL method.

### Teaching Plan

Recently, most Chinese universities have introduced online teaching platform, which can make up for the disadvantages of classroom instruction. Therefore, we used a combination of online educational platform (Chinese University MOOC) with CBL. This MOOC platform enables students to find learning resources that suits their needs and learn at their own pace.

In terms of curriculum design, the blended learning plan includes pre-class self-study, pre-class quizzes and face-to-face CBL classes. The pathophysiology course contents include water and electrolyte balance and imbalance, acid-base balance and imbalance, shock, hepatic failure, renal failure, heart failure, respiratory failure. Each lecture consists of two teaching hours (40 min each). (1) In pre-class self-study, students were required to read relevant chapters in the textbook and watch relevant videos on Chinese University MOOC platform. Meanwhile, the relevant PPT prepared previously by teachers and questions selected from the test database of Chinese Medical education were recommended by teachers to be used as Supplementary Materials. (2) During class, students completed a pre-class quiz (10–15 min, total 20 points) within the MOOC. Next, the teachers gave specific explanation for the questions with high incorrect answer ratios. (3) During the course of teaching, the teachers first presented the clinical cases to students. Then, students were asked to discuss a series of questions regarding the cases such as involved pathogenic mechanisms and therapeutic principles. A few student volunteers were asked to share their answers and received timely feedback from the teachers. (4) At the end of the course, the teachers summarized the main points of lessons and evaluated students' performances, as well as answer the questions.

### Course Assessment

All 2015, 2017, and 2018 class students were tested twice in the course: in the middle of course and at the end. The middle test included 60 multiple-choice questions with a total score of 60 points and conducted by means of an on-line examination. The final test was established in an offline, closed-book format. The examination papers with total 100 points were carefully prepared by experienced teachers, including multiple-choice questions (MCQ), short answer questions (SAQ) and case analysis questions. The papers had adequate coverage of the course contents.

### Questionnaire

To better assess this learning method we, respectively, conducted anonymous questionnaire survey to the students and teachers at the end of a term. The questionnaire was based on previous peer instruction studies in medical education (12, 13). The questionnaire determined students and teachers opinion regarding the blended learning method and Chinese University MOOC on a five-point Likert scale (1 = strongly disagree, 5 = strongly agree).

### Statistics

The quiz scores were presented as means ± standard deviation (SD). Data were analyzed using SPSS 12.0 software for Windows (SPSS Inc., Chicago, Illinois, USA). ANOVA, *T*-test and LSD were used to determine differences among 2015, 2017, and 2018 classes. For the results of the questionnaires, the collected data were analyzed using SPSS with Chi-square test. A *P*-value of <0.05 was considered statistically significant.

## Results

### Comparison of Student Achievement Under Different Learning Methods

**Figure 1:**
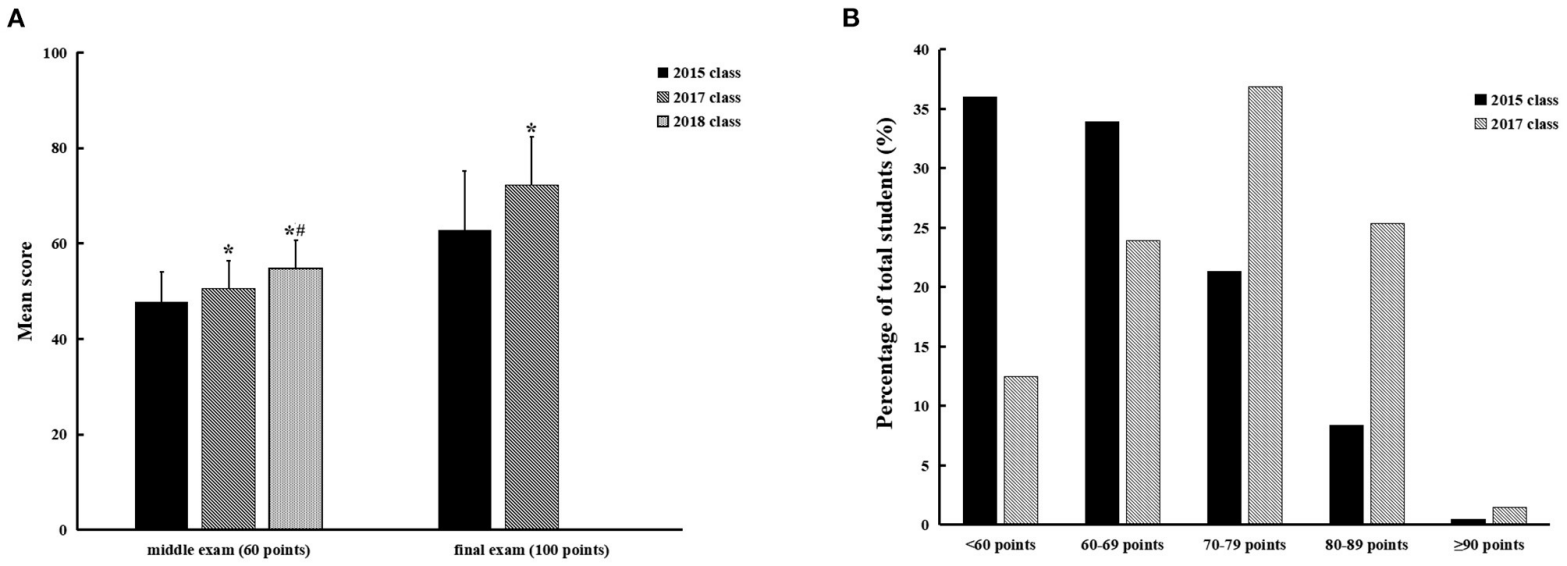
Comparison of student achievement between traditional instruction and blended learning. **(A)** Average scores of the middle exam and final exam in the 2015 class (*n* = 431), 2017 class (*n* = 418) and 2018 class (*n* = 586), analyzed by ANOVA, *T*-test and LSD. Values are means ± SD. *P < 0.001 vs. 2015 class without blended learning. ^#^*P* < 0.001 vs. 2017 class. **(B)** Distribution of the final exam scores in the 2015 class (*n* = 431) and 2017 class (*n* = 418). The results were presented as percentage of total students of the class.

The 2015 class took the pathophysiology course *via* traditional learning approach, whereas the 2017 and 2018 classes was taught using the blended learning method. Assessment of student performance in the middle of course showed that the mean exam score of the 2018 class (54.83 ± 5.76 points) was the highest, followed by the 2017 class (50.56 ± 5.85 points), while the mean score of the 2015 class (47.87 ± 6.11 points) was the lowest (see Figure 1A, *P* < 0.001). In the final exam for the 2018 class, the examination paper only include multiple-choice questions that were randomly selected from the Test Database of Chinese Medical Education maintained by People's Medical Publishing House. It was different from the final exam for the 2015 and 2017 classes. Thus, we only compared students' final exam performance of the 2015 and 2017 classes (Figure 1A). It was found that the mean score of the 2017 class with blended learning was statistically higher than that of the 2015 class (72.32 ± 9.98 points vs. 62.83 ± 12.28 points, *P* < 0.001). Meanwhile, we observed the distribution of the final exam scores (see Figure 1B). Student must score at least 60 points to pass the final exam. The proportion of students with a final score <60 points in the 2015 class was much higher than in the 2017 class (36.0 vs. 12.4%, *P* < 0.001). On the other hand, the proportion of students with a final score ≥ 80 points in the 2017 class (26.8%) significantly elevated in comparison with that in the 2015 class (8.8%) (*P* < 0.001).

### Observation of Students' Self-Study Before Class

**Figure 2:**
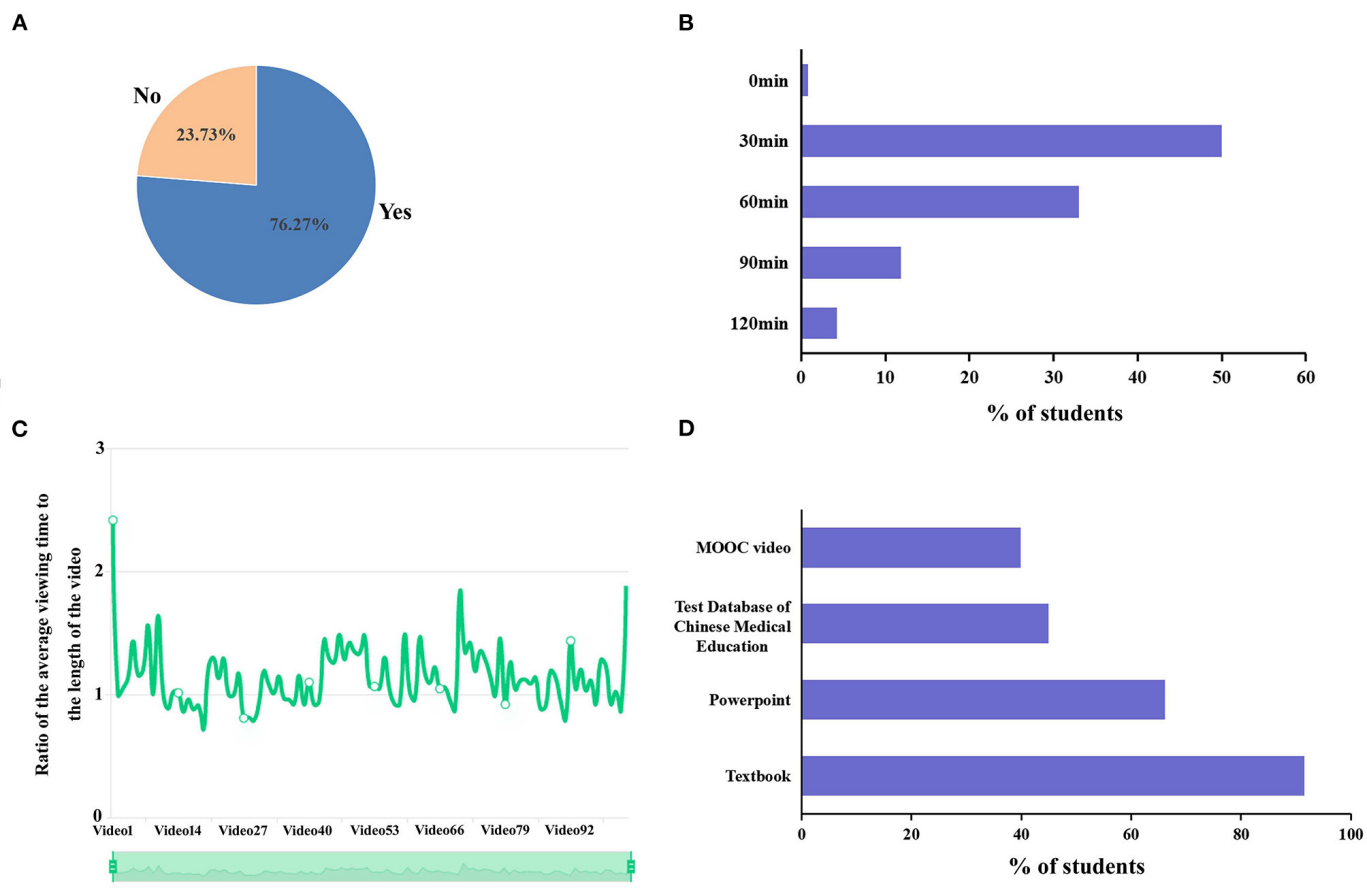
The status of students in 2018 class in completing the pathophysiology self-study before class (*n* = 118). **(A)** Percentages of students divided by whether they can complete the pre-view. **(B)** Time spent by students in pre-view. **(C)** Comparison of video viewing duration and video length (automatically generated by Chinese Universities MOOC platform). **(D)** Percentages of students who preferred different learning materials to complete the pre-learning.

In our teaching plan, the MOOC platform played an important role in pre-class self-study. With online videos shared on the platform, the students in 2017 and 2018 classes were required to preview the related content before class. The goal was to extend their learning time and improve the teaching effect. Students' pre-class quiz scores reflected that the majority of students really previewed the contents (Table 1). Meanwhile, to analyze students' self-learning activities with the MOOCs platform before class, the survey data were collected from a sample of 118 students in 2018 class using Wenjuanxing, an online survey platform. Totally, 76.27% students pre-viewed before class with the aid of the MOOC platform (Figure 2A). In terms of time, 33.05% students spent ~60 min completing the pre-class activities, while a half of students spent ~30 min on preview (Figure 2B). Hence, we were interested in how students watched the lecture videos in the online learning system. Since 2020, the Chinese University MOOC began to provide statistical data on students' online behaviors. The average viewing duration of each video is calculated, and then compared with the length of the video. For students in 2018 class, the data showed that the viewing time length of learners is generally 1–2 times longer than the length of the video, indicating that some students watch the same video repeatedly (Figure 2C). Nevertheless, regarding the learning materials for preview, the majority of students still preferred textbook as first-order preference (91.53%), followed by PPT (66.1%), the test database of Chinese Medical Education (44.92%) and MOOC videos (39.83%) (Figure 2D).

**Table 1:** Mean scores of pre-class quizzes in 2017 and 2018 classes (total 20 points).

**Class**	**Water and electrolyte balance and imbalance**	**Acid-base balance and imbalance**	**Shock**	**Hepatic failure**	**Renal failure**	**Heart failure**	**Respiratory failure**
2017 class	19.1 ± 1.3	16.6 ± 4.1	19.0 ± 1.9	18.8 ± 1.8	17.4 ± 4.8	17.0 ± 3.4	11.8 ± 3.9
2018 class	18.9 ± 1.8	17.9 ± 4.9	19.1 ± 1.8	19.9 ± 1.4	19.9 ± 1.8	19.8 ± 1.8	19.8 ± 1.2^*^

* *P < 0.05, Compared with corresponding 2017 class*.

### Student's Attitudes Toward Blended Learning

Students showed positive attitudes toward blended learning. They tended to express enjoyment and satisfaction from the blended learning experience (see Table 2). The majority of the 2017 class (69.9%) and 2018 class (67.8%) considered that blended learning meet the need of personalized learning, and the modified learning method helped 62.5% students of the 2017 class and 67.5% students of the 2018 class to manage their study time effectively. Moreover, 67% of students in the 2017 class and 66.4% of students in the 2018 class acknowledged that they took more initiative for learning in the blended format. When it comes to online resources for medical education, the majority of the students in both the 2017 and 2018 classes held the opinion that Chinese University's MOOC platform could provide appropriate and sufficient e-learning resources for students to conduct autonomous learning. However, it should be noticed that less students in the 2018 class agreed that the online resources of Chinese University's MOOC were suitable for their need in comparison with the 2017 class (55.0 vs. 66.5%, *P* < 0.05). Indeed, more students in the 2018 class chose “neutral” about these resources, indicating that they were not very satisfied with this platform.

**Table 2:** Responses from medical students regarding the blended learning experience.

**Item**	**Agree (score: 5 and 4)**				**Neutral (score: 3)**				**Disagree (score: 2 and 1)**			
	**2017 class** **(*****n*** **=** **176)**		**2018 class** **(*****n*** **=** **289)**		**2017 class** **(*****n*** **=** **176)**		**2018 class** **(*****n*** **=** **289)**		**2017 class** **(*****n*** **=** **176)**		**2018 class** **(*****n*** **=** **289)**	
	***n***	**%**	***n***	**%**	***n***	**%**	***n***	**%**	***n***	**%**	***n***	**%**
Chinese University's MOOC platform provides appropriate e-learning resources	117	66.5	159	55.0^*^	53	30.1	116	40.1	6	3.4	14	4.8
There are rich resources available for students on Chinese University's MOOC platform	120	68.2	213	73.7	49	27.8	62	21.5	7	4.0	14	4.8
The blended learning method met the need of personalized learning	123	69.9	196	67.8	44	25.0	73	25.3	9	5.1	20	6.9
The blended learning method enabled you to manage your study time effectively	110	62.5	195	67.5	49	27.8	72	24.9	17	9.7	22	7.6
The blended learning method enabled you to take more initiative for learning	118	67.0	192	66.4	50	28.4	76	26.3	8	4.5	21	7.3

*Values are n, no. of students, and percentage of students. Responses were scored using a 5-point Likert scale, ranging from 1 (strongly disagree) to 5 (strongly agree)*.

* *P < 0.05, Compared with corresponding 2017 class*.

### Teacher's Attitudes Toward Blended Learning

Analyzing teachers' questionnaire revealed that the majority of them expressed a willing to make use of Chinese University MOOC while designing a blended learning course (see Table 3). In addition, they considered that the combination of e-learning and CBL result in a positive effect on students' motivation, experience, performance and interaction with teachers. The teachers also believed that the implementation of blended learning made a transition from teacher-centered to student-centered mode of education, which was adapted to the requirement for cultivation of quality of talents. On the other hand, we observed that half of the teachers indicated a neutral response toward the online learning resources on Chinese University MOOC, which was consistent with students' response to this platform.

**Table 3:** Responses from the classroom teachers regarding the blended learning approach.

**Item**	**Agree (score: 5 and 4)**	**Neutral (score: 3)**	**Disagree (score: 2 and 1)**
The Chinese University MOOC-based blended learning helped to promote the research and practice of teaching innovation in pathophysiology	7	3	0
The Chinese University MOOC-based blended learning better met the demand for talent training in student-centered model	7	3	0
The Chinese University MOOC-based blended learning improved the quality of student learning	7	3	0
The Chinese University MOOC had online learning resources at an appropriate level for our students	5	5	0
The blended learning promoted the interaction between students and teachers	6	4	0
The blended learning met the need of personalized learning	9	1	0
The blended learning enabled students to manage their study time effectively	8	2	0
The blended learning enabled students to take more initiative for learning	7	3	0

*Values are no. of responses. Responses were scored using a 5-point Likert scale, ranging from 1 (strongly disagree) to 5 (strongly agree)*.

## Discussion

Traditional learning methods mainly consists of face-to-face classroom lectures where knowledge was passed from teacher to student *via* verbal interaction. However, students tend to remain relatively inactive or even passive, and depend on teachers (14). On the contrary, e-learning help student to achieve increased flexibility and give them more control over study pace. MOOC is a potent platform for open online resources, by which the learners can choose courses suitable for their learning needs. According to the data from the 2020 Global MOOC Conference held by Tsinghua University in September 2020, China ranks the first in the number of MOOCs (over 30 platforms and over 3,400 courses) (15). With the increasing number of education institutions that implement the online learning mode, MOOC is having a strong impact on the reform and development of Chinese higher education. In China, some national online platforms have appeared such as XuetangX, CNMOOC, Chinese University MOOC, “Fanya” and Zhihuishu. Liang et al. conducted a comparative analysis of these five platforms (16). The results showed that there are more effective discussion boards for communication in Chinese University MOOC platform, including general discussion board, comprehensive discussion board, teacher question-and-answer board and communication board, enhancing student-student and teacher-student interaction. Besides, the assessment criteria is only available in Chinese University MOOC platform, so that the learner can have proper evaluation about their learning level and plan their own learning. In particular, this platform has relatively complete course guidance resources in order to provide positive guidance for students with low self-learning ability. For these reasons, we used Chinese University MOOC as teaching support in our university where the students need more guidance and supervision from the teachers in comparison with students from well-known universities.

In our teaching plan reform, students preview course content in advance with open online courses from Chinese University MOOC. All online videos were selected by the pathophysiology team and watched by the students at least once, which provides learners with in-depth explanations and valuable repetition. Generally, most students could complete autonomous learning before class, and spent <60 min in pre-viewing. After the preview with online courses, students gained a general understanding of the main course contents. Next, in the face-to-face classes, we adopted CBL as a teaching method other than conventional lecturing. Studies have shown that CBL promotes deep learning and better understanding of the contents. Moreover, CBL helps student to improve critical thinking, analytical and problem solving skills (17). It is worth mentioning that students in local universities exactly lack these skills in some extent because of inadequate cultivation of creative and practical ability. As a result, the undergraduate courses of these universities tend to produce a qualified medical graduate, but not an educated man who will become more and more qualified as time progresses by lifelong learning. Therefore, we believed that with the implementation of CBL in regular curriculum, students could not only enhance their understanding of the course content but promote critical thinking and creativity abilities after the classes ended. Simultaneously, in the process of CBL course, students' attitudes toward the subject shifted from “what I have been taught” to “what I want to learn,” where they were prompted by teachers to learn the knowledge that was relevant in order to solve the problem (18). As expected, most of the students enrolled in the blended pathophysiology courses agreed that the learning method cultivated their autonomous learning abilities.

It is well-accepted that successful implementation of CBL is based on proper case selection and question design (level appropriate for students). In the context of relatively low professional ability and comprehensive quality, each of the “cases” and “problems” was carefully designed in close consultation with clinicians and experienced CBL faculty. The “cases” covered all the key and difficult points of the course. Conversely, the easy parts required students to study by themselves, allowing more class time for discussion. What's more, the “problems” mainly focused on general principle instead of specific technical measures, and thus most of students could find out correct answers to the questions after consulting literature and discussing with other students. This learning method enabled students to cultivate their clinical interests and increase their confidence in themselves.

Students' performance under the blended learning approach was, respectively, evaluated in the middle of course and at the end of course. The middle exam was online and included only multiple-choice questions. We found that performance of the 2018 and 2017 classes was both better than that of the 2015 class. Unlike the middle exam, the final exam was closed book and more complex, including MCQ, SAQ and case analysis questions. Thus, the final test score could further reflect student learning performance. More profound differences were found with respect to average exam scores between the 2015 and 2017 classes. Moreover, the proportion of students scoring in the 80s and 90s in the 2015 class was lower than that in the 2017 class, and more students in the 2015 class failed to pass the final exam (whose score was lower than 60 points). On the basis of these results, it can be concluded that the blended learning, combination of Chinese University MOOC approaches with CBL, promotes the learning of student and improves their academic performance. Meanwhile, given the higher mean score of the 2018 class than 2017 class, it may be speculated that students' performance could further arise as this blended learning method becomes more flexible and controllable.

Since the launch of Chinese University MOOC in 2014, this platform provides learners with high-quality open online courses, the number of which grew rapidly (19). For example, the online courses of pathophysiology were only offered by Central South University in 2019, while in 2020, three more well-known Chinese universities begin to provide the public with MOOC courses including Huazhong University of Science and Technology, NanChang University and Wuhan University. Considering the high quality of course content on Chinese University MOOC platform, we made innovation on teaching mode of pathophysiology based on this MOOC. To understand students' attitude toward Chinese University MOOC-based blended learning course in pathophysiology, a total of 465 questionnaires from students (176 in the 2017 class and 289 in the 2018 class) were collected and analyzed. The blended learning approach had a good acceptance from the students. The majority of the students agreed that the most useful advantage of the blended learning model is time flexibility and location convenience. Because of this, they could effectively control the time and pace of their learning, finally promoting their learning autonomy.

Unexpectedly, we observed that students in the 2018 class had less positive attitude toward Chinese University MOOC in comparison with students in the 2017 class, although more students acknowledged that this platform provided rich medical resources. This was consistent with the survey in which students in 2018 class tend to use textbooks and PPT rather than MOOC videos for pre-class study. Chinese University MOOC is famous for the top-quality courses offered by well-known universities, thus these courses are designed primarily for the students of high ability in these institutions (20). Nevertheless, the students' professional ability and comprehensive quality is relatively low in local/regional institutions such as Guilin Medical University (21). As a result, some courses contain insufficient detail for our students, while others are really hard to understand against the background of students' low level of prior knowledge. In this context, we tried to integrate Chinese University MOOC teaching resources with self-built resources including videos, PPT and chapter tests available on campus network. The resources of self-built courses were carefully designed by pathophysiology teaching team according to the medical licensing examination requirements, and more closely related to our students' level of knowledge and ability. This change was implemented from the start of the 2018 class, which might partly contribute to the increased average score of the middle test in comparison with the 2017 class. Therefore, we will continue to integrate high-quality online resources such as Chinese University MOOC into our blended learning in pathophysiology courses at Guilin Medical University in the future teaching practice. On the other hand, the proportion of our self-built online resources may gradually increase.

We also collected questionnaire data from the involved teachers. Generally, they were satisfied with Chinese University MOOC-based blended learning and welcomed this student-centered learning method model. In agreement with students' opinion, most teachers believed that the blended learning was a good way to motivate students and cultivate their ability of autonomous learning and communication, leading to improved students' performance in pathophysiology. Hereby, they would desire to continue the implementation of blended learning in teaching pathophysiology. Otherwise, a half of teachers were “neutral” on the applicability of Chinese University MOOC as an independent learning platform, triggering the further development of self-built online resources.

Taken together, it is concluded that blended learning method that combined Chinese University MOOC with face-to-face CBL courses could improve students' performance and motivation. This learning method was valued positively by the students and teachers for its flexibility and personalization, and suitable for the talent training in local universities. Meanwhile, as a result of this research, it became clear that teachers in local medical universities urgently need to better identify and use more appropriate online learning resources that match students' ability and academic levels.

## Data Availability Statement

The original contributions presented in the study are included in the article, further inquiries can be directed to the corresponding authors.

## Ethics Statement

The studies involving human participants were reviewed and approved by Ethics Committee of Guilin Medical University. The patients/participants provided their written informed consent to participate in this study.

## Author Contributions

JT and HL conceived and designed research and approved final version of manuscript. HL, QW, and XL performed experiments. GS analyzed data. DW, HL, and XL interpreted results of experiments. JT prepared figures. JZ drafted manuscript. JT, DW, and HL edited and revised manuscript. All authors contributed to the article and approved the submitted version.

## Funding

This work was supported by Guangxi Higher Education Teaching Reform Projects (No. 2017JGB328, 2018JGZ130), Innovation Project of Guangxi Graduate Education (No. JGY2019160), Guangxi Scholarship Fund of Guangxi Education Department, and Guilin Medical University Teaching Research and Reform Program (No. JG202003).

## Conflict of Interest

The authors declare that the research was conducted in the absence of any commercial or financial relationships that could be construed as a potential conflict of interest.

## Publisher's Note

All claims expressed in this article are solely those of the authors and do not necessarily represent those of their affiliated organizations, or those of the publisher, the editors and the reviewers. Any product that may be evaluated in this article, or claim that may be made by its manufacturer, is not guaranteed or endorsed by the publisher.
